# Long-Term Monitoring of the Seasonal Abundance of *Basidiobolus* spp. in Gecko Feces in KwaZulu-Natal (South Africa)

**DOI:** 10.3390/jof8090943

**Published:** 2022-09-07

**Authors:** Maike Claussen, Stefan Schmidt

**Affiliations:** Discipline of Microbiology, School of Life Sciences, University of KwaZulu-Natal, Pietermaritzburg 3201, South Africa

**Keywords:** *Basidiobolus*, abundance, gecko, seasons, thermotolerant, climate, South Africa

## Abstract

The fungal genus *Basidiobolus* is typically associated with ectothermic animals such as amphibians and reptiles. In rare cases, it can cause infections in humans, which are often misdiagnosed. Although usually restricted to tropical and subtropical countries, infections have recently been more frequently reported in hot-dry regions such as Arizona and Saudi Arabia. Reptiles such as geckos are known to shed *Basidiobolus* spp. via feces and frequently live in close proximity to humans. To establish the frequency and burden of *Basidiobolus* spp. released by geckos in a suburban location, we regularly quantified viable *Basidiobolus* units per gram of feces from indoors and outdoors over 3.5 years between 2018 and 2022 using a selective medium. Geckos were shedding *Basidiobolus* spp. in all seasons, with most counts established ranging between 5.0 and 6.5 log_10_ cfu per gram. Statistically significant seasonal differences per location were only observed for the outside winter counts and, apparently, correlated to lower temperatures, while inside counts showed no seasonal difference. Overall, counts for droppings collected outdoors were significantly higher than counts for droppings collected indoors. Our data confirm that geckos, which frequently enter homes and are global invaders, are a regular source of this fungus.

## 1. Introduction

*Basidiobolus* species are filamentous fungi that can be found in various plant- and animal-derived environmental samples and have been reported from all continents, with the exception of Antarctica. Originally isolated from frogs by Eidam in 1886, the association of *Basidiobolus* spp. with especially reptiles and amphibia is well established [1,2,3,4,5,6,7,8]. Certain members of the genus *Basidiobolus* can cause rare infections in mammals, including humans, usually confined to tropical and subtropical regions [9,10]. Infections are often misdiagnosed and mostly affect the skin and subcutaneous tissues; however, intestinal infections have been reported [9,10,11,12,13,14,15,16]. Susceptibility to basidiobolomycosis is not limited to immune compromised humans like many other fungal diseases but is more often reported to affect healthy and younger individuals [9,10,11,15,16]. The pathway of infection is still not clearly established, but minor trauma (“toilet leaves”, insect bites) are assumed to be the entry port for subdermal infections, while ingestion of contaminated food or soil might lead to gastrointestinal basidiobolomycosis (GIB) [9,10,14,16,17,18,19]. As geckos are frequently present inside homes, their small fecal droppings, which were previously shown to contain *Basidiobolus* spp. [20], can be accidentally consumed (e.g., with food) and might pose a health risk. However, an infectious dose has not yet been evaluated and confirmed, making it difficult to identify infection routes and predict possible risk doses.

Basidiobolomycosis is an emerging fungal disease reported in the USA and the Middle East, with systemic infections occurring even in hot and dry areas such as Arizona and the Arabic peninsula [11,13,14,15,16,18,21]. This might be due to the occurrence and emergence of more infectious strains and, additionally, due to better diagnostics and awareness of such rare diseases [14,16,22]. It is important to identify and characterize potential fungal pathogens, but, even more so, it is crucial to understand the fungal diversity and ecology of both pathogenic and non-pathogenic strains. Climate change with rising temperatures is assumed to contribute to an increase in fungal pathogenicity and the occurrence of fungal infections [23,24]. Moreover, numerous microbial diseases are known to occur typically in specific seasons. Climate, local weather and geographical area determine the markedness of different seasons. In turn, the different seasons can affect the presence and abundance of microbial pathogens in environments, hosts or vectors [25]. However, such seasonality will differ for each pathogen and is affected by various factors such as geographic location, climate and local weather, occurrence and behavior of vectors, predisposition and living conditions of hosts [25].

The presence of *Basidiobolus* spp. in different environmental samples of plant and animal origin is well known, but quantitative data are sparse, and the seasonal distribution and current abundance of this fungal genus has been poorly investigated. It was reported that *Basidiobolus* spp. can be easily isolated from frogs, but not when the animals hibernate during the colder season [1,3]. However, in warmer climates, amphibia and reptiles are active the whole year. Geckos, which are known vectors of microbial pathogens [26,27], are commonly associated with human dwellings and are expanding their distribution globally by invading new regions [28,29]. Additionally, the shedding of *Basidiobolus* spp. by reptiles is assumed as a possible source of infection [4,5,7,30]. Data on the geographical and seasonal abundance of pathogens is crucial when assessing potential health risks for the public. Accordingly, analyzing the distribution and abundance of the potentially pathogenic fungal genus *Basidiobolus* in the environment is important for epidemiological assessments that require data on possible sources, vectors and seasonal variations in frequency and abundance. 

Pietermaritzburg, the capital of the province of KwaZulu-Natal in South Africa, has marked seasonal weather differences and was recently identified as a town in South Africa potentially experiencing dangerous heat stress levels [31]. Additionally, geckos are commonly present in houses. Therefore, we undertook long-term monitoring to investigate if *Basidiobolus* spp. is present in outdoor and indoor gecko droppings collected in all four meteorological seasons and how many viable *Basidiobolus* units (VBUs) are detectable. Furthermore, we analyzed if selected climate factors affected VBUs present in gecko droppings.

## 2. Materials and Methods

### 2.1. Sampling

A total of 242 fecal samples from geckos (*Hemidactylus* spp., the only reptiles observed on the house walls) (Figure S1) were collected on 87 occasions over 3.5 years between September 2018 and February 2022 in a suburban area of Pietermaritzburg, South Africa. Corresponding samples were taken from outside locations (close to the house wall on pavements and windowsills below the eaves, typically near outside light posts) and inside locations (typically on windowsills, shelves or on the wall) (Figure S1). Samples, usually consisting of 10 droppings, were incrementally taken from each location within the same sampling period. The collection date and period depended on the occurrence and availability of inside droppings. If possible, two samples were taken per location and sampling period. In cases where no droppings could be collected from the inside for an extended period of time, samples were collected only from the outside to ensure seasonal coverage. Each sampling occasion was assigned to the corresponding meteorological season of the southern hemisphere. For the outside locations, 144 samples were collected on 86 sampling occasions, and from the inside, 98 samples were collected on 81 sampling occasions. All samples were stored in sterile collection tubes at ambient temperature until analysis (typically within 1 week after the last collection day of the sampling period). 

Due to the COVID-19 pandemic lockdown restrictions implemented in South Africa, access to the laboratory was not available from 20 March 2020 to 12 September 2020 and 18 December 2020 to 26 February 2021. During this time, samples were collected as usual and stored in sterile collection tubes at ambient temperature inside a Styrofoam rack until access to the lab was permitted again.

### 2.2. Enumeration

For the quantification of viable *Basidiobolus* units (VBUs), samples were initially diluted 10-fold in sterile saline, thoroughly mixed for 20–30 min and further decimally diluted, typically up to 10^−4^. Of each dilution, 100 µL was spread-plated onto Rose Bengal Chloramphenicol agar supplemented with Dichloran and Benomyl and incubated at 28 °C for 4–6 days as previously described [20]. Simultaneously, the presence or absence of VBUs in the gecko fecal samples was verified using the canopy method as described by Coremans-Pelseneer [4] with the following modification: a portion of the initially diluted sample was placed on a sterile filter paper prewetted with Nutrient broth, placed in the lid of a Petri dish containing Sabouraud Dextrose agar and incubated upside-down as described above. Representative colonies were confirmed using microscopy and random isolates were confirmed using genus specific PCR as described previously [20]. Counts of VBUs were calculated using the weighted mean approach and provided as colony forming units per gram of gecko fecal matter (cfu/g) and transformed to log_10_ cfu/g, if required. The absence of *Basidiobolus* spp. in samples was reported as 1 cfu per g to enable log transformation. 

For correlation analysis, average counts were established for each sampling occasion if more than one sample was available for the specific location.

### 2.3. Climate Data

Daily data of temperature (min/max °C), relative humidity (min/max %) and rainfall (mm) were sourced for Pietermaritzburg from the South African Weather Service (SAWS, weather station climno 0239698 5). Missing data points were substituted using weather data kindly provided by Dr. Alistair Clulow (UKZN, School of AEES), sourced from the weather station situated on the Ukulinga research farm (Pietermaritzburg). For each sampling occasion, the following climate-related characteristics were calculated for the corresponding sampling period:

- Number of days with a maximum temperature of >30, <20, <15 °C
- Number of days with a minimum temperature of >15, <10, <5 °C
- Number of days with a maximum relative humidity >95, <80%
- Number of days with a minimum relative humidity >80, <50%
- Number of days with rain ≥1 mm (wet days)
- Number of days with rain ≥10 mm (substantial rain)
- Total precipitation during the sampling period (in mm).

### 2.4. Statistical Analysis

Descriptive and comparative statistics were established using Microsoft Excel (MSO Professional Plus 2016) and the statistical add-in software tool Analyse-it v6.01.1 for Excel (Leeds, UK). Single counts were used to compare all inside and outside samples and to analyze possible seasonal effects/differences. Normality of data distribution was tested using the Shapiro—Wilk test. Non-parametric tests were applied for comparisons; the Wilcoxon—Mann—Whitney method was used for comparing two groups and the Kruskal—Wallis test for comparing more than two groups. The Steel—Dwass—Critchlow—Fligner method was applied as post-hoc test for multiple all-pairs of groups comparisons. Average counts per sampling occasion (where applicable) were used for climate correlation analysis. The Spearman rank correlation test was used to assess possible associations and correlations between selected climate factors and counts. A *p*-value of <0.01 was selected as a minimum acceptance threshold for significance.

## 3. Results

**Figure 1:**
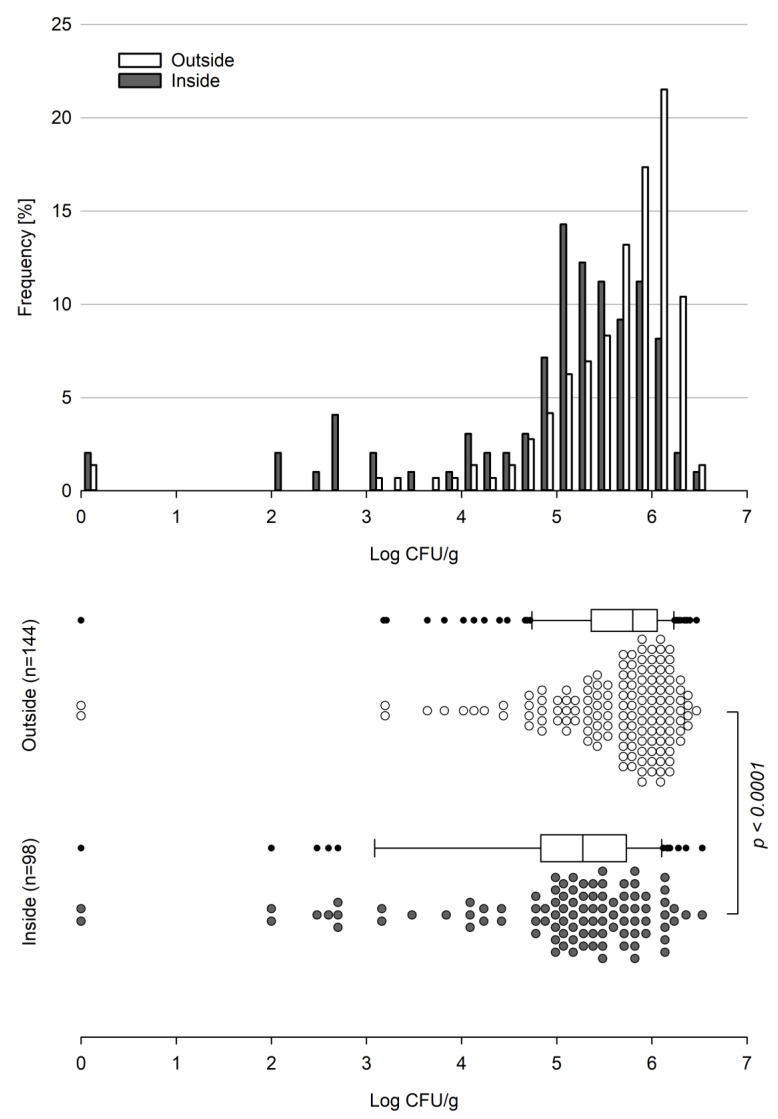
Frequency and range of viable *Basidiobolus* spp. counts obtained over 3.5 years in gecko droppings collected from inside and outside locations. The *p*-value was calculated using the Wilcoxon—Mann—Whitney test.

*Basidiobolus* spp. proved to be present in gecko fecal samples throughout all seasons between September 2018 and February 2022. The majority (>75%) of all 242 samples analyzed showed counts above 5.0 log_10_ cfu per gram of gecko droppings with maximum counts of 6.47 and 6.53 log_10_ cfu/g for the outside and inside, respectively. With the exception of two samples with no detectable viable *Basidiobolus* units (VBUs) present, all counts determined for the outside were larger than 3.0 log_10_ cfu/g, and about 64% (92 out of 144) of the outside samples showed log counts of ≥5.6 log_10_ cfu/g, sharply peaking in the range of 6.0 to 6.2 log_10_ cfu per gram (Figure 1). 

As for the outside, two samples from the inside tested negative for *Basidiobolus* spp.; however, the majority of inside samples showed a broad count distribution starting from 2.0 log_10_ cfu/g and peaking in the range of 5.0 to 5.2 log_10_ cfu/g, while only about 32% (31 out of 98) of the samples showed counts above 5.6 log_10_ cfu per gram of gecko droppings.

Overall, counts established for the outside (n = 144) significantly differed (*p*-value < 0.0001) from counts established for the inside (n = 98) (Figure 1), with a mean of 770,081 (5.89 log_10_) cfu/g for the outside and 406,072 (5.61 log_10_) cfu/g for the inside. The corresponding median values were 627,000 (5.80 log_10_) cfu/g and 189,000 (5.28 log_10_) cfu/g for the outside and inside samples, respectively. Considering the low average weight of a fecal gecko dropping (12 and 16 mg for inside and outside samples, respectively), this corresponds to an estimated average burden of about 12,300 cfu per outside and about 4800 cfu per inside gecko dropping.

Although samples collected during the lockdown periods in 2020/21 (autumn and winter 2020, and summer 2020/21) had to be stored for an extended period of time before analysis could take place (up to 176 days in the first lockdown), no statistically significant differences in counts for the outside data and most inside data were detectable when compared with data from corresponding seasons without lockdown restrictions (Table S1). Only inside samples from the beginning of the first lockdown period (autumn season 2020) showed a statistically significant difference compared to corresponding autumn seasons (Table S1), which coincided with the fact that people were restricted in their movement and had to stay at home. However, with 709,000 cfu/g the count established for the earliest indoor sample taken in the first lockdown in autumn 2020, which was stored for the longest time, was higher than counts established for inside samples taken later in the same lockdown period.

**Figure 2:**
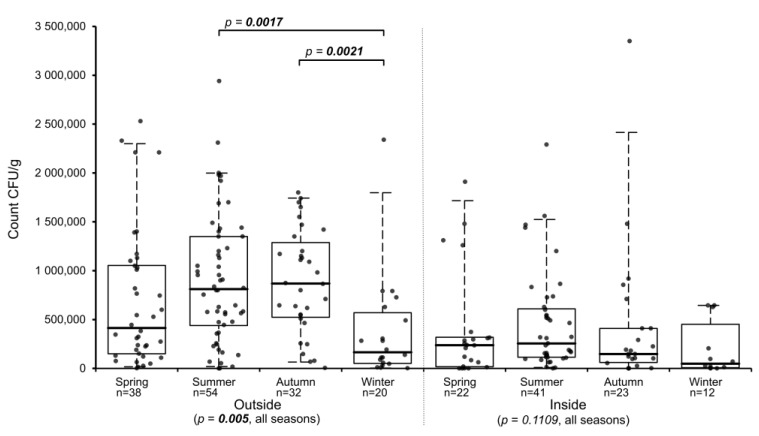
Seasonal comparison of viable *Basidiobolus* spp. counts established for inside (left hand) and outside gecko droppings (right hand). *p*-values below compare counts of all seasons per location using the Kruskal—Wallis test. *p*-values above show only significant values obtained using the post hoc test (Steel—Dwass—Critchlow—Fligner) comparing all seasonal pairs per location. Statistically significant differences are marked in bold.

To determine if seasonal differences exist for the abundance of *Basidiobolus* spp. in gecko feces, all samples were assigned to the corresponding seasons by their sampling periods separated by location (indoors, outdoors). Samples from the inside showed no statistically relevant differences between all four seasons (*p*-value = 0.1109), while outside samples from the various seasons differed significantly (*p*-value = 0.005). In summer and autumn, outside samples had the highest median counts, while samples collected in winter had the lowest median and those collected in spring had an intermediate median count (Figure 2). Multiple all-pairs of groups comparison analysis of the seasonal distribution of *Basidiobolus* spp. in gecko feces for outside samples revealed that only the winter seasons differed statistically from the summer and the autumn seasons (Table 1 and Figure 2), while inside samples showed no significant differences between any of the four seasons (Table 2). When comparing the counts from matching seasons of outdoor and indoor samples, statistically significant differences existed between the two locations in summer and autumn (Table S2).

**Figure 3:**
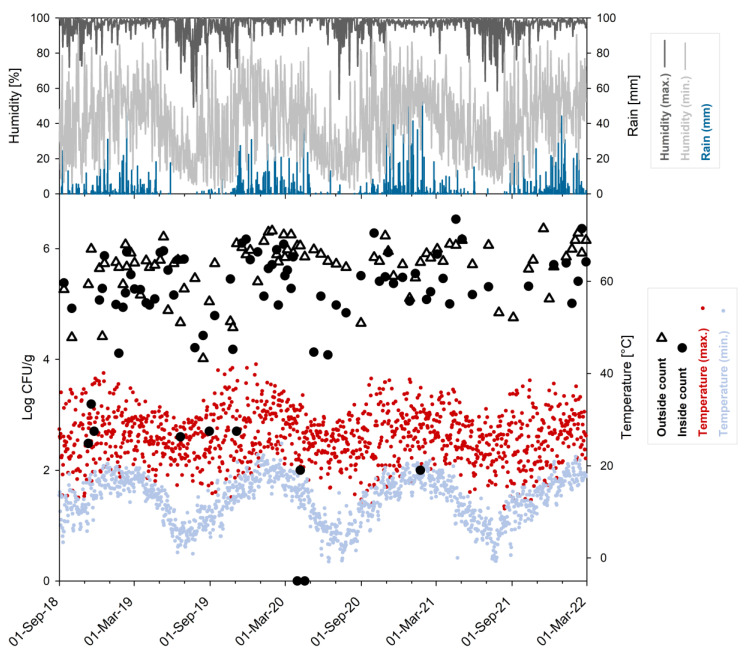
Daily climate data (rain, humidity and temperature) and average viable *Basidiobolus* spp. counts (indoor and outdoor) observed over 3.5 years of monitoring.

The distribution of average counts from inside and outside locations in relation to the daily climate data (total rainfall, minimum and maximum values for temperature and relative humidity) obtained for the sampling period of 3.5 years is shown in Figure 3. 

Similar to the established single count data, average counts from the outside (n = 86, log_10_ cfu/g: mean 5.87, median 5.79) were statistically different at 1% significance level when compared to the average counts established for inside locations (n = 81, log_10_ cfu/g: mean 5.61, median 5.28) (Figure S2).

Pietermaritzburg is located in the subtropical zone of the southern hemisphere at an altitude of about 600 m. Differences between the four meteorological seasons are apparent but less prominent than in temperate climate zones like northern Europe. Although light frost can occur at night in winter, daily temperatures are usually more moderate; yet, the minimum temperature in winter was always below 15 °C within the monitored period of 3.5 years. The monthly average minimum temperature ranged from about 5–9 °C in the winter months to about 16–19 °C in summer (Table S3). On the other hand, the monthly average maximum temperature was always higher than 22 °C for all seasons. However, days with a maximum temperature below 20 °C did occur, mainly in winter and spring, while days above 30 °C were scarce in winter but frequent in mid-summer. Days with a maximum temperature below 15 °C were never observed in summer and only once in autumn (May 2021) within the 3.5 years of monitoring. 

To analyze a possible correlation between climate-related factors and the abundance of *Basidiobolus* spp. in gecko droppings, average counts per sampling location (if applicable), temperature, humidity and rainfall data were determined for the corresponding sampling periods.

**Figure 4:**
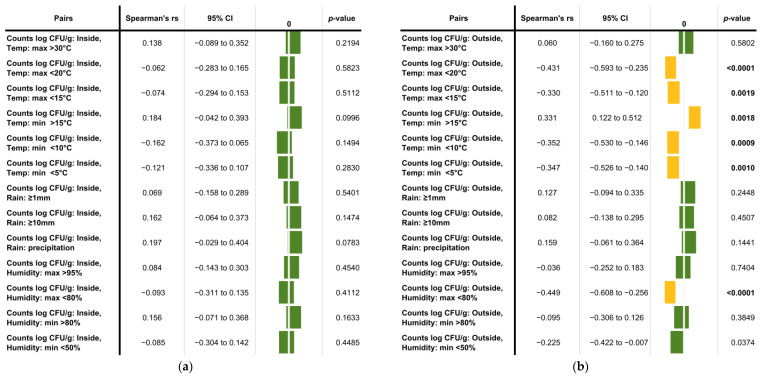
Correlation analysis between average viable *Basidiobolus* spp. counts and selected climate data for: (**a**) Gecko droppings collected indoors and (**b**) gecko droppings collected outdoors. Statistically significant correlations are highlighted in yellow with *p*-values marked in bold.

Based on the obtained data, the climate parameters evaluated in this study had no significant effect on the average counts established for inside samples (Figure 4a). However, a moderate, statistically significant correlation was observable between temperature and the *Basidiobolus* spp. burden in outside gecko droppings. Lower maximum (<20 and <15 °C) and minimum temperatures (<10 and <5 °C) affected the burden negatively, while a minimum temperature above 15 °C had a positive influence on the *Basidiobolus* counts in gecko droppings collected outdoors (Figure 4b). Additionally, a maximum relative humidity below 80% showed a negative impact on the *Basidiobolus* burden in outside droppings, while rain had no statistically significant effect (Figure 4b).

## 4. Discussion

Our study demonstrates that throughout all the seasons of the year *Basidiobolus* spp. is regularly shed by geckos via feces. More than 95% of the indoor and outdoor gecko droppings analyzed tested positive for *Basidiobolus* spp., confirming data obtained in a previous study [20]. However, in contrast to our observations, earlier studies from tropical Africa reported a frequency of about 10 to 15% of *Basidiobolus* spp. in gecko derived samples [4,5]. Unfortunately, quantitative data for *Basidiobolus* spp. in animal feces or other environmental samples are still sparse. Meanwhile, gecko feces analyzed in our current study carried on average a *Basidiobolus* burden of 7.7 × 10^5^ cfu per g for outdoor and 4.1 × 10^5^ cfu per g for indoor samples. This is in line with previously reported results from South Africa, where nearly all samples of gecko droppings carried *Basidiobolus* spp. with positive samples showing counts between 300–1.4 × 10^6^ cfu per g feces [20]. Interestingly, the previous study indicated a lower *Basidiobolus* burden in droppings from inside than outside locations, which was also evident in the present long-term monitoring study. Indeed, the VBUs in samples collected indoors proved to be significantly lower than the VBUs in samples collected outdoors. Furthermore, the proportion of samples with counts of ≥5.6 log_10_ cfu per gram of feces was two-fold higher for samples from the outside (64%) than from the inside (32%). Geckos are territorial and typically occupy microhabitats [32], and usually smaller and fewer geckos were observed indoors (personal observation). However, geckos can freely enter and leave houses through cracks, vents or open windows (Figure S1). This might explain why gecko droppings still contain considerable amounts of *Basidiobolus* spp. albeit the indoor environment represents a less ideal microhabitat regarding prey availability. Additionally, the presence of homeowners will affect the behavior and presence of indoor geckos. Due to the strict regulations implemented in the first lockdown period of the COVID-19 pandemic in 2020 (20 March to 12 September), citizens in South Africa were not allowed to leave home during the implemented curfew without permission, except for food supply or medical reasons. This resulted in a changed domestic routine of homeowners on the premise where the sampling took place and, additionally, delayed the analysis procedure as access to the laboratory was prohibited. Yet, our results indicate that the long-term storage of gecko feces at ambient temperature had no significant effect on the viable *Basidiobolus* units present in gecko feces, as counts for outdoor samples did not significantly differ from counts established in matching non-lockdown samples. Low counts determined for indoor samples of gecko droppings, especially in autumn 2020, are, therefore, more likely a result of the sudden permanent presence of people inside the house, which in turn might have disturbed the inside gecko population and changed their behavior, retreat and/or food availability. Furthermore, in the colder winter months, the number of active geckos in the evening visibly declined. This is not unexpected as the activity of ectothermic animals usually decreases with declining temperatures, resulting in decreased food uptake. If less and maybe different prey is available, it can affect the presence of *Basidiobolus* spp. in the gut and feces of geckos. Frogs captured during or at the end of hibernation (a period without food uptake) were reported to carry no viable *Basidiobolus* spp., while amphibia tested in summer and autumn usually did [1,3]. It was additionally reported that reptiles and frogs kept in the laboratory lose *Basidiobolus* spp. within about 1 to 3 weeks [2,3,6], indicating that the type of food or lack of food might play a role in the occurrence of *Basidiobolus* spp. in the feces of these animals. Indeed, the *Basidiobolus* burden found in gecko droppings is a reflection of the burden present in the intestines of the reptiles. 

Although *Basidiobolus* spp. was present in gecko droppings in all four seasons in our study, outdoor winter droppings were less frequently available and showed significantly lower counts compared to samples collected in summer and spring. A possible explanation for this kind of seasonality could be that the *Basidiobolus* population present in the geckos declines in winter (due to lower food uptake and temperature), recovers in spring and culminates again during the warmer months in summer and autumn. Moreover, counts established for gecko feces collected outdoors and indoors did not significantly differ in winter and spring. A possible explanation is that in colder months, outside geckos might temporarily retreat to locations that are protected from low temperatures, such as areas inside houses. Such behavior was reported for Mediterranean geckos in Oklahoma (USA), where geckos kept partially active by using heated buildings as a winter retreat [33]. Geckos are ectothermic and their body temperature is influenced by the outside temperature. Choosing warmer retreats could be a way for geckos to stabilize and maintain their body temperature during colder periods. A recent study from Australia analyzed the mean body temperature (T_b_) of the Asian house gecko *Hemidactylus frenatus* and showed that geckos from tropical locations (Thailand) maintained a T_b_ within a narrow range of 30 to 33 °C throughout the year [34]. The body temperature of the same gecko species from temperate southeastern Australia, however, was lower in winter (25–26 °C) compared to summer (about 29 °C) and was additionally influenced by feed uptake, which resulted in an increase of about 2–3 °C after feeding [34]. Given the similar annual climate profiles, it is likely that geckos in Pietermaritzburg exhibit a body temperature response similar to the Australian geckos, with lower outside temperatures resulting in a decrease in body temperature. Considering the high number of viable *Basidiobolus* spp. present in gecko feces, it is highly likely that *Basidiobolus* spp. is not only passing the digestive system of geckos but is able to replicate in the gut before it is shed, which was previously suggested for agama lizards [4]. Thus, the body temperature of geckos will affect the growth of *Basidiobolus* spp. present in the intestines. Our data indicate that higher maximum temperatures had no significant effect on the counts determined in gecko feces, which is not surprising as the mean body temperature of geckos is reported to be maintained in summer and in warmer climates at about 29 to 33 °C [34], and all members of the genus *Basidiobolus* are known to grow well at such temperatures [5,8,35,36,37,38]. On the other hand, previous studies indicated that strains of *Basidiobolus* spp. differ in their growth temperature preferences and not all can grow well at low temperatures [35,38,39,40]. The correlation analysis indicated that the significantly lower *Basidiobolus* spp. counts observed in winter were linked to clearly lower daily temperatures. The number of days with lower minimum (<10, <5 °C) and maximum (<20, <15 °C) temperatures negatively affected the established *Basidiobolus* spp. counts in gecko droppings. These findings coincide with fewer geckos and their droppings present indoors and outdoors in winter reflecting dormancy or a reduced activity of herptiles in colder periods due to lower body temperature and possibly reduced food intake. In turn, this will slow down or even disrupt the growth of *Basidiobolus* spp. in the intestines of geckos. On the other hand, a minimum temperature above 15 °C, which likely allows geckos to maintain a higher body temperature, had a positive influence on the fecal *Basidiobolus* burden. This indicates the need of a certain minimum temperature for sufficient growth and the presence of certain *Basidiobolus* strains in the gut system of hosts or vectors like geckos. 

Interestingly, a low maximum humidity of <80% produced a statistically significant correlation and negatively impacted the *Basidiobolus* counts in outdoor gecko feces. This is somewhat unexpected considering reports of emerging intestinal *Basidiobolus* infections from arid areas such as Arizona. However, the occurrence of a maximum humidity below 80% coincides with times of low temperatures; the correlation might therefore reflect the influence of low temperature. Rain on the other hand had no significant impact despite the fact that in Pietermaritzburg the majority of rainfall usually occurs during summer while winter months are dryer and less humid (usually less than three wet days per month). However, outdoor samples were collected from areas that are partially protected from light rain (close to the house under the eaves), while heavy rain would have destroyed or washed the droppings away. Similarly, gecko droppings inside buildings are protected from rain and, to some degree, from other extreme climate-related factors (for example, the indoor temperatures never dropped below 10 °C). This could also explain why climate-related correlations were not apparent in samples collected indoors. 

The results of the correlation analysis indicate that the climate does contribute to seasonal variations observed for *Basidiobolus* spp. counts in gecko dropping samples. However, it can only partially explain the differences, as individual climate factors with significant correlations showed only a limited contribution. This is not unexpected as seasonal variations are driven by a complex network of varying abiotic and biotic environmental factors influencing the community that is living in an ecosystem. Climate conditions such as temperature and precipitation are prominent and easily measurable factors, though effects might be indirect or even delayed. Additionally, other factors, such as interactions with the co-existing microflora and the host/vector population, behavior, health and diet, will also affect the *Basidiobolus* spp. abundance. As mentioned above, the food or diet of small reptiles (which usually consists of insects and other invertebrates) seem to play a role in the presence of *Basidiobolus* spp. in their gut system [2,3,6]. 

Although survivability was not tested in the present study, the results obtained during the lockdown indicate that *Basidiobolus* spp. counts were not significantly affected by long-term storage. It can therefore be assumed that *Basidiobolus* spp. can survive an extended period of time in dry gecko droppings, matching previous reports of dryness survival [2,3,41].

Difficulties in isolating *Basidiobolus* spp. from animals and their dung in winter were reported before [1,3,39]. Previously, we observed a lower *Basidiobolus* spp. burden for gecko droppings during winter [20], which was confirmed in the present study. Similarly, a study from India reported a higher detection frequency for *Basidiobolus* spp. from the intestines of bats captured during the summer months in the area of Delhi and New Delhi [42]. The climate pattern of Delhi and New Delhi is comparable to the climate pattern in Pietermaritzburg with a hot and wet summer period and a cooler and dryer winter period, although average temperatures are slightly lower in Pietermaritzburg. These reported differences in the detection of *Basidiobolus* spp. from small animals in subtropical areas with comparably mild winters still indicate that a certain seasonality seems to exist. Unfortunately, annual frequency and quantitative distribution data of *Basidiobolus* spp. in possible reservoirs such as small animals are rare, especially for regions where basidiobolomycosis occurs more often.

It is still puzzling why basidiobolomycosis only occurs in certain, usually more tropical regions, albeit this fungal genus being globally present in various environments. Although our data clearly indicate that considerable amounts of *Basidiobolus* spp. are shed by geckos, so far, only one case of subcutaneous basidiobolomycosis has been reported for South Africa [43]. As infections with *Basidiobolus* spp. are not limited to immune suppressed people but affect healthy and young humans, it is less likely that the health of the population determines susceptibility. While exposure or subclinical infections might cause immunity in the population, underlying deficiencies, such as gastric acid repression, were discussed as possible factors enhancing susceptibility [9,16,18]. Underdiagnosis and possible misdiagnosis of fungal infections as a result of less attention given to human mycoses are additional challenges, especially in Africa [44]. Even though fungi account worldwide for many infections, surveillance and epidemiological investigations of mycoses and studies on the causing agents are still underrepresented compared to viral and bacterial infections [24,44]. Importantly, it is known that the pathogenicity and virulence can differ between closely related fungal species or strains [24], which might similarly apply to the genus *Basidiobolus*. Additionally, the proportion of pathogenic to non-pathogenic strains inhabiting different vectors or areas can vary and, therefore, affect the occurrence of diseases. Several species of the genus *Basidiobolus* have been linked to infections [9,10,19,21], but a prerequisite for causing infections in animals and humans is the ability to grow at body temperatures. Not surprisingly, clinical *Basidiobolus* isolates were mostly reported to be more thermotolerant with optimum growth at body temperatures and many even able to grow at 40 °C [35,36,45]. This physiological parameter is currently not used for *Basidiobolus* species differentiation and characterization, although thermotolerance can contribute to higher infectiousness in fungi [23,24]. The different temperature preferences of *Basidiobolus* strains could also relate to the season and climate of their geographic origin, which was previously suggested [39,40]. Thermotolerant and thus possibly more pathogenic strains are likely to be more abundant in warmer climates, while cold-tolerant *Basidiobolus* strains could tend to dominate in colder climates and might be less infectious.

In a previous study we showed that *Basidiobolus* spp. can be distinguished by their different preferred growth temperature profiles, mainly based on their ability to grow at lower (6 °C) or higher (40 °C) temperatures [38]. Preliminary data analyzing the temperature profiles of gecko fecal isolates collected outdoors suggest that the *Basidiobolus* population in gecko feces from Pietermaritzburg is dominated in summer by strains able to grow at 40 °C (Figure S3). Notably, the proportion of cold-tolerant isolates (able to grow well at 6 but not at 40 °C) seemed to increase in winter. This indicates that the proportion of different *Basidiobolus* spp. strains present in geckos might change depending on season or climate (e.g., temperature). However, this observation is based on a limited number of samples and requires confirmation. Furthermore, it would be important to investigate if such variations in the proportion of different thermally adapted *Basidiobolus* isolates can also be found in other environments or geographical regions and if this is linked to *Basidiobolus* infections.

Climate change and increasing global temperatures could promote adaptation to higher temperatures. The emergence of *Candida auris* as a novel global pathogen is currently hypothesized to be caused by global warming causing rising temperatures [23,24]. Additionally, adaptation to higher temperatures could also change host or vector specificity, which in turn could contribute to the global distribution of such pathogens, for example, by migrating birds. Many small animals, such as geckos, are also well-known invaders, which are often spread via global shipping routes [28,29]. A recent study from Saudi Arabia showed that *Basidiobolus* isolates from gastrointestinal infections were genetically identical to isolates obtained from the common house gecko (or Asian house gecko, *Hemidactylus frenatus*) [30]. This gecko species originates from tropical Asia and is, like the dominating gecko species in South Africa *Hemidactylus mabouia*, a well-known global invader [28,29]. If such geckos regularly carry potentially pathogenic strains of *Basidiobolus* spp., this might contribute to the worldwide distribution of basidiobolomycosis. As a precaution, in regions where geckos commonly enter houses and are present in the kitchen area, surfaces and places where food is prepared should be regularly inspected for the presence of fecal gecko droppings and prepared food should be protected from contamination when left outside (for example, by covering it).

## 5. Conclusions

Our data show that considerable amounts of viable *Basidiobolus* units are present in local gecko feces in all four seasons. Climate correlation analysis indicated that lower temperatures reduced the number of viable *Basidiobolus* units in gecko fecal droppings. However, more data representing different climates and geographic regions are necessary to confirm these correlations. Considering climate change in the context of globally rising temperatures, the abundance of thermotolerant *Basidiobolus* strains should be monitored. Additionally, to enable epidemiological predictions, investigations on the quantitative distribution, pathogenicity, virulence and host specificity of *Basidiobolus* spp., especially in regions where basidiobolomycoses are reported, would be essential.

## Figures and Tables

**Table 1:** Multiple comparisons (Steel—Dwass—Critchlow—Fligner post hoc test) of seasonal *Basidiobolus* spp. counts in gecko droppings collected from the outside.

Outside	Spring (*p*-Value)	Summer (*p*-Value)	Autumn (*p*-Value)	Winter (*p*-Value)
**Spring**		0.2134	0.1714	0.1497
**Summer**	0.2134		0.9919	**0.0017**
**Autumn**	0.1714	0.9919		**0.0021**
**Winter**	0.1497	**0.0017**	**0.0021**	

Statistically significant *p*-values are marked in bold.

**Table 2:** Multiple comparisons (Steel—Dwass—Critchlow—Fligner post hoc test) of seasonal *Basidiobolus* spp. counts in gecko droppings collected from the inside.

Inside	Spring (*p*-Value)	Summer (*p*-Value)	Autumn (*p*-Value)	Winter (*p*-Value)
**Spring**		0.6517	0.9920	0.6674
**Summer**	0.6517		0.5397	0.0919
**Autumn**	0.9920	0.5397		0.6163
**Winter**	0.6674	0.0919	0.6163	

## Data Availability

Data are contained within this article and the supplementary material.

## Supplementary Materials

The following supporting information can be downloaded at: https://www.mdpi.com/article/10.3390/jof8090943/s1, Figure S1: Appearance of geckos and their droppings in typical inside and outside locations; Figure S2: Comparison of average counts established for gecko droppings collected from the inside and outside locations over 3.5 years using the Wilcoxon—Mann—Whitney test; Figure S3: Proportion of cold-tolerant, intermediate and thermotolerant *Basidiobolus* strains isolated from gecko droppings collected in the summer and the winter season; Table S1: Statistical analysis of the lockdown effect on *Basidiobolus* spp. counts in gecko droppings collected in different seasons; Table S2: Statistical comparison of *Basidiobolus* spp. counts of indoor and outdoor samples from the matching seasons; Table S3: Monthly average temperatures for Pietermaritzburg (01/2018–02/2022).
