# Supplementary material for: Long-Term Monitoring of the Seasonal Abundance of Basidiobolus spp. in Gecko Feces in KwaZulu-Natal (South Africa)
Source: J Fungi (Basel). 2022 Sep 7;8(9):943. doi: 10.3390/jof8090943 (PMC9506373; doi:10.3390/jof8090943)
Supplement: Supplementary file 1 [file jof-08-00943-s001.zip › jof-1865678-supplementary.pdf]

Supporting information (Long-term monitoring of the seasonal abundance of *Basidiobolus* spp. in gecko feces in KwaZulu-Natal (South Africa), Claussen & Schmidt 2022)

Table S1. Statistical analysis of the lockdown effect on viable *Basidiobolus* spp. counts in gecko droppings collected in different seasons.

Seasonal pairs	Indoor samples (*p*-value)	Outdoor samples (*p*-value)
**Autumn counts: With lockdown, Autumn counts: No lockdown**	**0.0067**[1]	0.7941[2]
**Winter counts: With lockdown, Winter counts: No lockdown**	1.0000[1]	0.2703[1]
**Summer counts: With lockdown, Summer counts: No lockdown**	0.0567[1]	0.0378[1]
**Spring counts:**	Not affected by any lockdown	

[1] Wilcoxon-Mann-Whitney test
[2] Students t-test (two-sided)
Statistically significant *p*-values are marked in bold

Table S2. Statistical comparison of viable *Basidiobolus* spp. counts of indoor and outdoor samples from the matching seasons.

Pairs	*p*-value[1]
**Autumn: Indoor counts, Autumn: Outdoor counts**	**0.0003**
**Winter: Indoor counts, Winter: Outdoor counts**	0.1338
**Summer: Indoor counts, Summer: Outdoor counts**	**0.0002**
**Spring: Indoor counts, Spring: Outdoor counts**	0.0406

[1] Wilcoxon-Mann-Whitney test
Statistically significant *p*-values are marked in bold

Table S3. Monthly average temperatures for Pietermaritzburg (01/2018-02/2022).

	**Monthly average maximum / minimum temperatures (°C)**										
	**2018**		**2019**		**2020**		**2021**		**2022**		
Month	max	min	max	min	max	min	max	min	max	min	Seasons
**January**	**(29.0)**	**(16.7)**	**27.5**	**16.7**	**30.4**	**19.1**	**28.5**	**17.1**	**28.7**	**17.6**	**Summer**
**February**	**(28.7)**	**(17.1)**	**28.2**	**16.7**	**29.8**	**18.5**	**27.9**	**17.3**	**28.1**	**18.3**	**Summer**
**March**	**(27.4)**	**(15.8)**	**27.8**	**16.9**	**29.2**	**16.1**	**28.2**	**16.2**			**Autumn**
**April**	**(25.9)**	**(14.5)**	**24.8**	**14.0**	**25.6**	**13.6**	**28.0**	**13.0**			**Autumn**
**May**	**(23.7)**	**(9.4)**	**25.8**	**10.7**	**25.5**	**8.0**	**25.3**	**9.4**			**Autumn**
**June**	**(23.2)**	**(6.0)**	**24.2**	**5.5**	**23.9**	**5.2**	**23.6**	**7.2**			**Winter**
**July**	**(22.8)**	**(5.1)**	**25.5**	**5.3**	**24.8**	**4.6**	**22.8**	**3.7**			**Winter**
**August**	**(22.4)**	**(8.2)**	**24.8**	**9.3**	**24.4**	**6.5**	**24.1**	**7.8**			**Winter**
**September**	**24.9**	**10.7**	**26.5**	**9.6**	**25.3**	**11.0**	**25.1**	**11.4**			**Spring**
**October**	**25.6**	**10.7**	**28.8**	**12.8**	**26.6**	**14.1**	**23.9**	**11.4**			**Spring**
**November**	**26.5**	**13.1**	**27.5**	**15.8**	**26.7**	**15.1**	**24.9**	**14.2**			**Spring**
**December**	**28.5**	**17.0**	**27.0**	**16.5**	**28.4**	**17.3**	**26.6**	**16.0**			**Summer**
**Annual low**	**11.5**	**-0.1**	**13.2**	**0.9**	**11.6**	**-0.8**	**10.7**	**-0.7**			
**Annual high**	**40.1**	**20.4**	**42**	**22.4**	**38.9**	**24.8**	**38.5**	**21.1**			

Values in brackets are data from outside the monitoring period of the study

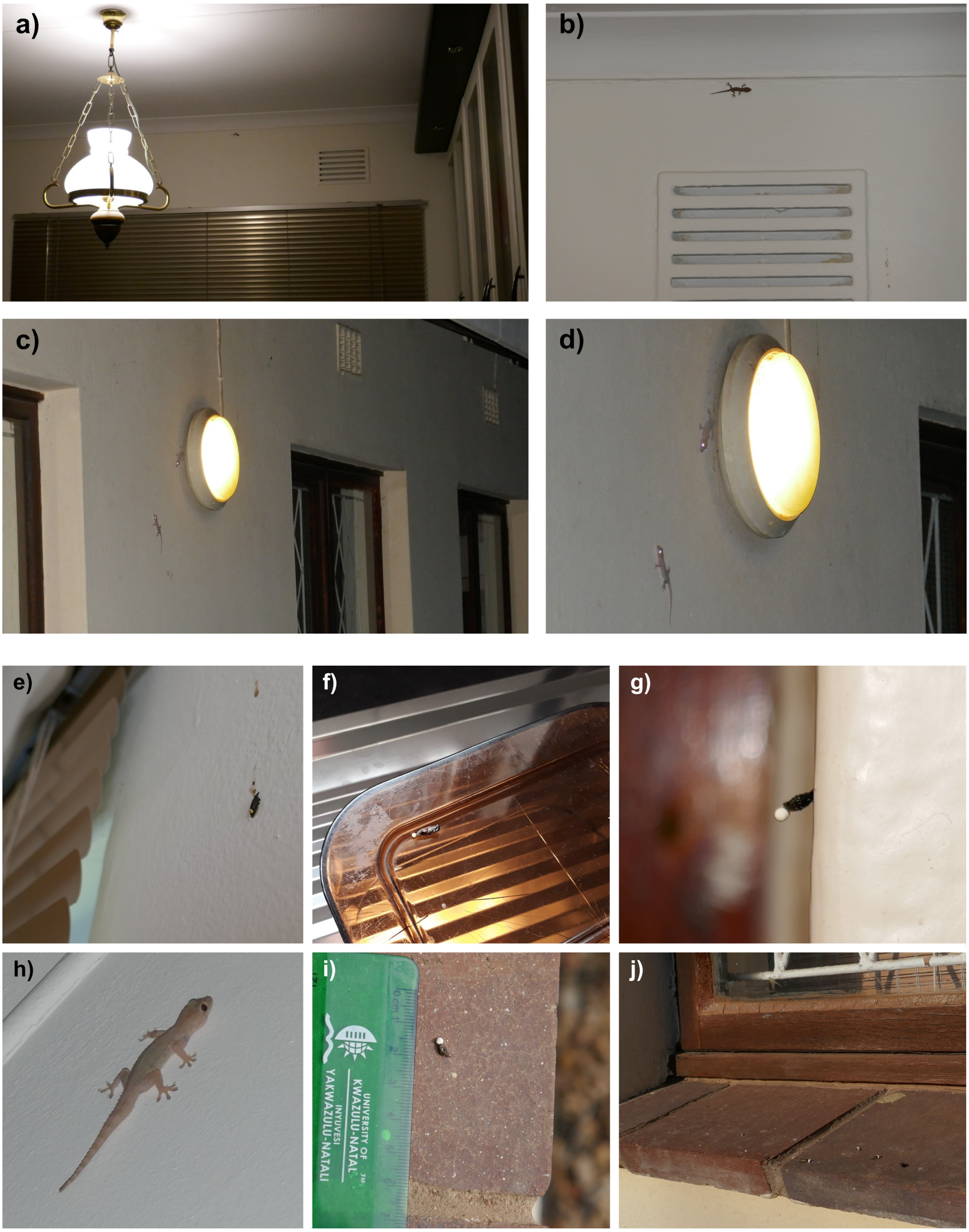

Figure S1: Appearance of geckos and their droppings in typical inside and outside locations.
a) and b) inside locations;
c) and d) outside locations;
e), f), g) examples of gecko indoor droppings;
h) representative gecko specimen (*Hemidactylus* sp.) on the house wall;
i) and j) examples of outdoor gecko droppings.

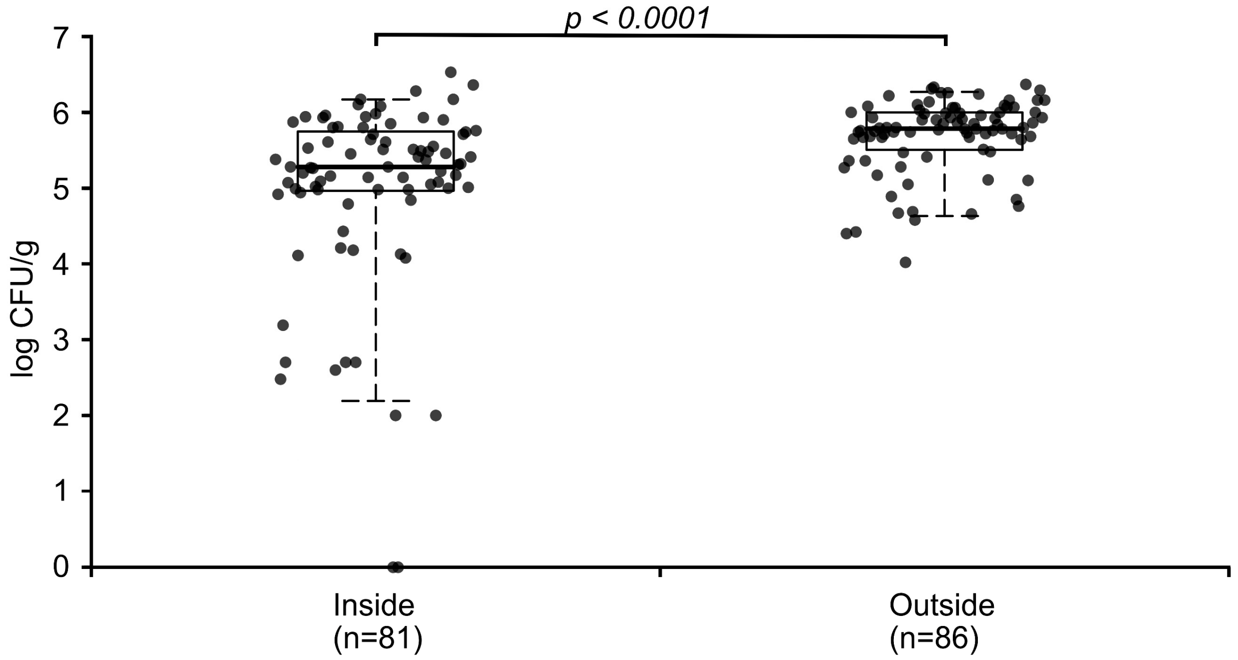

Figure S2: Comparison of average viable *Basidiobolus* counts established for gecko droppings collected from the inside and outside locations over 3.5 years using the Wilcoxon-Mann-Whitney test.

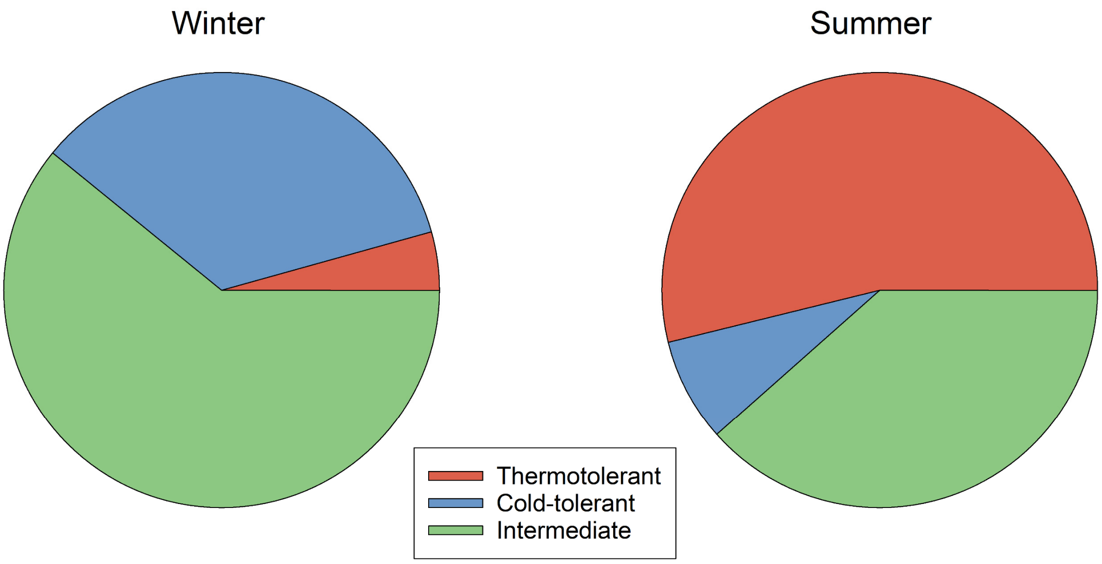

Figure S3: Proportion of cold-tolerant, intermediate and thermotolerant *Basidiobolus* strains isolated from gecko droppings collected in the summer and the winter season.
Randomly selected isolates (summer n=26 from 3 sampling occasions and winter n=23 from 2 sampling occasion) were analyzed for their ability to grow at 6 and 40 °C on Sabouraud agar and assigned to one of the following groups [39]:

- Thermotolerant: no growth at 6 °C, good growth at 40 °C.
- Cold-tolerant: good growth at 6 °C, no growth at 40 °C.
- Intermediate: growth at 6 °C and growth at 40 °C.
